# Effect of Licorice (*Glycyrrhiza glabra*) Extract as an Immunostimulant on Serum and Skin Mucus Immune Parameters, Transcriptomic Responses of Immune-Related Gene, and Disease Resistance Against *Yersinia ruckeri* in Rainbow Trout (*Oncorhynchus mykiss*)

**DOI:** 10.3389/fvets.2022.811684

**Published:** 2022-02-23

**Authors:** Mostafa Darvishi, Mehdi Shamsaie Mehrgan, Amir Eghbal Khajehrahimi

**Affiliations:** ^1^Department of Fisheries, Science and Research Branch, Islamic Azad University, Tehran, Iran; ^2^Department of Food Science and Technology, North Tehran Branch, Islamic Azad University, Tehran, Iran

**Keywords:** bactericidal activity, cytokines, disease resistance, herbs, innate immunity

## Abstract

This study was designed to appraise the effect of licorice herbal supplement on the immune status of rainbow trout fingerlings. Accordingly, five diets were formulated with different levels of licorice extract (LE) including 0 (control), 0.5 g kg^−1^ (LE0.5), 1 g kg^−1^ (LE1), 2 g kg^−1^ (LE2), and 3 g kg^−1^ (LE3). The fingerlings (10.0 ± 0.1 g initial mean weight) received the diets in triplicates (30 fish in each replicate) for 56 days. The results showed that the white blood cells and their differential number (lymphocytes and monocytes) were remarkably increased by LE2 supplementation (*P* < 0.05). The oral administration of LE2 significantly increased the levels of serum immunoglobulin (Ig), lysozyme activity, and complement components (C3 and C4) compared with others. Meanwhile, the serum bactericidal activity against *Yersinia ruckeri* in LE2 and LE3 treatments was significantly higher than others except for LE1 (*P* < 0.05). In addition, serum alternative complement activity significantly improved in all treated groups except LE0.5 compared with the control group (*P* < 0.05). In terms of skin mucosal immunity, the fish fed with LE2 and LE3 diets exhibited notably higher lysozyme activity, alkaline phosphatase activity, and Ig value than other groups (*P* < 0.05). The highest skin mucus bactericidal activity against *Y. ruckeri* was obtained in LE2 treatment (*P* < 0.05). In addition, dietary LE2 significantly increased the relative expression of immune-associated genes including *tumor necrosis factor-*α, *interleukin-1*β, *interleukin-8*, and *IgM* and the former treatments showed higher values than the control group. The cumulative mortality of fish against *Y. ruckeri* infection was notably reduced from 53.6% in the control group to 29.0% in LE3 treatment. Overall, the dietary administration of LE at 2 g kg^−1^ had the best effects on immunocompetence in rainbow trout.

## Introduction

Over the past decade, the global production of rainbow trout (*Oncorhynchus mykiss*) has grown significantly and nearly doubled. However, the immune system of fish can be suppressed by various stressors in captivity conditions, and the emergence of infectious diseases has hampered the growth performance and survival rate of rainbow trout in aquaculture sectors (1). It has been widely proven that the improvement of aquafeed plays a chief role in fish health status (2–4). Therefore, a healthy balanced diet should not only be formulated based on the essential requirements of aquatic animals but should also contain some ingredients to ameliorate the overall performance of fish in captivity (5–7). In this regard, several functional feedstuffs such as acidifiers, probiotics, and medicine plants with diverse biological activities such as antioxidant and immune-boosting properties have entered into aquaculture nutrition (8–11). In recent years, there has been increasing attention to the use of herbal supplements in aquaculture due to their high potency (i.e., availability, relatively low price, easy processing, and eco-friendly) (8–11). In fact, herbal supplements display immunostimulatory effects on the host due to several bioactive compounds that are unique for each medicinal plant species (12).

Licorice, *Glycyrrhiza glabra*, is a medicinal plant belonging to the Fabaceae family and native to the Mediterranean, southern Russia, and Asia. In addition, it is cultivated throughout Europe, the Middle East, and Asia (13). The roots are the most important part of the plant, which is widely used in the pharmaceutical, health, and food industries (13, 14). Flavonoids, glycyrrhizinic acid (glycyrrhizin), glabridin, liqueurite, and liqueurizine are the main biomolecules of the roots, which have potential antimicrobial, anti-inflammatory, antihepatotoxic, antimutagenic, and antioxidant effects (14–17). Recent studies have found that dietary licorice roots can boost the non-specific immune system in several fish species, such as common carp, *Cyprinus carpio* (18), and Nile tilapia, *Oreochromis niloticus* (19). Moreover, other findings showed that licorice root powder enhances hepatoprotective and anti-stress effects in aquatic animals (20, 21). Herbal extract supplements have become more common in aquaculture nutrition than botanical powders due to higher absorption rates, higher bioactive compounds, and lower dietary dosage to meet the medicinal benefits. As with most plant extracts, licorice root extract has more potency and consistency than the powder form due to higher bioactive phytochemicals, especially glycyrrhizin (22). However, the information about the dietary effects of licorice root extract on immunological defense mechanisms in fish or shellfish is scarce. Therefore, in this research, the influence of dietary licorice extract (LE) was evaluated for the first time on the immune system (skin mucus and serum immune responses), the expression of related genes, and *Yersinia ruckeri* resistance in rainbow trout as one of the most widely introduced species in the finfish aquaculture industry.

## Materials and Methods

### Plant Hydro-Alcoholic Extraction Process

The dried and cleaned rhizomes and roots of licorice (*G. glabra*) were prepared from Dineh Company, Tehran, Iran. Then, they were pulverized into a fine powder (0.2 mm) using a grinder. Then, the powder (300 g) was soaked in 70% ethanol (1.5 L) at 4°C, and the mixture was stirred periodically. After 72 h, the mixture was filtered by Whatman No. 1 paper. The solvent (ethanol) was removed by vacuum rotary method at 90 rpm at 50°C. A final 50 ml of concentrated extract was obtained from 300 g of dried rhizomes. Finally, the extract was dried at room temperature and then stored in a refrigerator until use (23).

### Experimental Diets

The basal diet (42.5% protein, 16.4% fat, and 9.6% ash) was prepared by mixing the dry ingredients including 42% fish meal, 20.8% soybean meal, 16.8% wheat flour, 6% Kilka fish oil, 4% soybean oil, 2% mineral premix, 2% vitamin premix, 1% cellulose, 0.3% phytase, 0.1% DL-methionine, and 0.2% antioxidant. To make five experimental diets, different levels of LE including 0 (control), 0.5 g kg^−1^ (LE0.5), 1 g kg^−1^ (LE 1), 2 g kg^−1^ (LE 2), and 3 g kg^−1^ (LE 3) along with 250 ml of distilled water were mixed with the feed stuffs for each experimental group. In the next step, the prepared paste was turned into pellets using a meat grinder (Pars Khazar, Tehran, Iran; 3-mm die), and then, the produced pellets were dried at room temperature using a conventional fan until their moisture content reached below 10%.

### Experimental Fish

In this study, 500 rainbow trout fingerlings were purchased from a private farm in Firuzkuh (Tehran, Iran) and transferred alive to the Khojir Research Station (Tehran, Iran). The fish health was checked by examining their physical appearance and behavior such as skin color, swimming style, eye and gill status, abnormalities in the spine, fin rot, and external parasitic contaminations. Before the start of the feeding trial, the fish were stored for 2 weeks in circular tanks (1,000 L) to acclimate to new conditions (water temperature of 15.0–15.8°C, dissolved oxygen of 7.5–8.5 mg L^−1^, and pH 7.2–7.8), and they were fed with the basal diet. At the end of 14 days, 450 rainbow trout (mean weight ± SD; 10.0 ± 0.1 g) were distributed in 15 square concrete tanks (700 L; 30 fish in each tank) with a 0.3 L s^−1^ water flow rate. During the rearing period, feeding was done three times a day (8:30, 12:30, and 16:30) on the basis of apparent satiety for 56 days.

### Blood and Epidermal Mucus Sampling

At the end of the rearing period, the fish were deprived of feed for 24 h and three fish were randomly caught from each tank and then anesthetized with clove powder (150 mg L^−1^). Then, 2 ml of blood was taken from each rainbow trout using a syringe from the caudal vein. To measure hematological parameters, a part of the individual blood sample was transferred to a heparinized tube. The other part was transferred to a non-heparinized tube and kept in the refrigerator for 4 h (for clotting) and then centrifuged at 3,000 ×*g* for 15 min at 4°C. The collected supernatants (serum) were stored at −80°C for further analysis.

To measure mucosal immune parameters, three fish were randomly caught from each tank and individually placed in polyethylene bags containing 2 ml of chloride sodium (50 mM). The fish was gently rubbed for ~2 min, the skin mucus was collected and centrifuged at 1,500 ×*g* for 10 min, and then the supernatants were transferred to sterile tubes and kept at −80°C for further experiments (24).

### Blood and Skin Mucus Immune Parameters

White blood cells (WBCs) were calculated using a hemocytometer slide on the basis of the method of Martins et al. (25). In addition, the differential count of WBC including neutrophils (Neu), lymphocytes (Lym), monocytes (Mono), and eosinophils (Eos) was performed by preparing and staining the blood smear according to the method recommended by Borges et al. (26) under an optical microscope (Alphaphot-2 YS2, Nikon, Japan). The total protein (TP) values of the skin mucus and serum samples were estimated using the method described by Lowry et al. (27).

Total immunoglobulin (Ig) of the serum or skin mucus sample was measured according to the method previously recommended by Siwicki and Anderson (28), in which the amount of Ig was calculated by subtracting the protein concentration of the sample before and after the addition of polyethylene glycol.

The serum or skin mucus lysozyme (LYZ) activity was measured according to the protocol described by Ellis (29) using the turbidimetric method. For this purpose, 50 μl of the serum or skin mucus sample was mixed with 2 ml of bacterial suspension along with citrate-phosphate buffer (0.2 mg ml^−1^ in a 0.05 M sodium phosphate buffer, pH 6.2). Then, the rate of reduction of light absorption of each sample up to 15 min at 450 nm was measured by an ELISA reader (800 TS, BioTek Instruments Inc., USA). The amount of LYZ activity in each serum or mucus sample was calculated using the standard egg white LYZ curve (Sigma-Aldrich).

Serum alternative complement activity (ACH50) was measured on the basis of the method described by Yano (30) using sheep red blood cell hemolysis. The volume yielding 50% hemolysis was estimated and, in turn, used for quantifying the complement activity of the serum. In addition, the serum complement components (C3 and C4) and skin mucus alkaline phosphatase (ALP) were measured using the relevant commercial diagnostic kits for fish (Hangzhou Eastbiopharm Co., Hangzhou, China) by a clinical automated blood analyzer (Prestige 24i, Tokyo Boeki, Japan) as previously used for rainbow trout (31). Serum and mucus bactericidal activity (BA) against *Y. ruckeri* (KC291153; isolated from infected rainbow trout and maintained at the Faculty of Veterinary Science, University of Tehran, Iran) was evaluated according to the method described by Fazelan et al. (32). In brief, the bacterium was cultivated in a nutrient broth, washed, and suspended in phosphate-buffered saline (PBS). The optical density of the bacterial suspension was adjusted to 0.5 at 600 nm (33). This suspension was serially diluted five times (1:10) by PBS. Afterward, 20 μl of the serum and 100 μl of the mucus were added to 2 μl of the bacterial suspension and incubated at 22°C for 1 h. In addition, PBS (20 μl) was used as the control. Finally, the samples were cultured on trypticase soy agar medium at 22°C for 24 h, and the grown colonies on the plates (in triplicates) were counted to determine BA against *Y. ruckeri* as log_10_ colony-forming unit (CFU) ml^−1^.

### Gene Expression Study

To evaluate the expression of the immune-related gene, three fish were randomly taken from each tank, and after euthanasia with clove powder, the anterior kidney was removed on ice and immediately stored at −196°C in sterile tubes. In the Genetic Laboratory of Zakariya-Razi Complex Center (Science and Research University, Tehran, Iran), 100 mg of the head kidney was ground, transferred to sterilized tubes, and subjected to the total RNA extraction process *via* an RNX-Plus kit^®^ (SinaClon Co., Tehran, Iran) according to the manufacturer's instructions. Besides, DNase (Invitrogen, USA) was used to remove possible genomic DNA in the extracted RNA. The extracted RNA was stored at −80°C until the synthesis of cDNA. For this purpose, a cDNA synthesis kit (GeNet Bio Co., Daejeon, South Korea) was used. Accordingly, 2 μl of the template RNA was transferred to a 0.2-ml tube, and oligonucleotide reagents and primers were added. Then, the mixture was incubated at 44°C for 60 min. In the next step, the reaction was subjected to 75°C for 5 min to inactivate the cDNA degrading enzymes. The volume of solution containing the synthesized cDNA was adjusted to 20 μl (34).

Quantitative real-time PCR was carried out using a SYBR Green Master Mix kit (GeNet BIO Inc., Daejeon, South Korea) in a real-time thermal cycler (Rotor-Gene Q, QIAGEN, Germany). The thermal profile for the reactions consists of 2 min at 94°C for initial denaturation; then 40 cycles for 30 s at 94°C, 30 s at 62°C, and 45 s at 72°C for annealing temperature; and one cycle for 5 min at 70°C for the final extension.

The intensity of fluorescence at the end of each cycle was recorded by the qPCR device (Rotor-Gene Q). β*-actin* gene was used as a reference gene to normalize the expression of the target genes. The primers used in this experiment were designed using Primer3 online software version 0.4 (Table 1). The relative expression of candidate genes in different treatments was performed on the basis of the 2^−Δ*ΔCt*^ method.

**Table 1:** Sequences of the oligonucleotide primers used in this study for real-time qPCR^*^.

**Gene**	**Primer sequences**	**Efficiency**	**Accession**
	**(F/W forward and reverse primers)**	**(%)**	**number**
*TNF-α*	F: GGGGACAAACTGTGGACTGA	98.0	AJ277604.2
	R: GAAGTTCTTGCCCTGCTCTG		
*IgM*	F: ACCCTCCTCTTGGTCGTTTC	96.7	S63348
	R: TGATGACACCAACAGCAACA		
*IL-1 β*	F: ACATTGCCAACCTCATCATCG	97.1	AJ223954.1
	R: TTGAGCAGGTCCTTGTCCTTG		
*IL-8*	F: AGAATGTCAGCCAGCCTTGT	96.6	AJ279069
	R: TCTCAGACTCATCCCCTCAGT		
*β-actin*	F: ACATCAAGGAGAAGCTGTGCTAC	97.8	AB196465
	R: TACGGATGTCCACGTCACAC		

* *TNF-α, tumor necrosis factor α; IgM, immunoglobulin M; IL-1β, interleukin-1β; IL-8, interleukin-8*.

### *In vivo* Fish Infection Model

After the feeding trial, the resistance of the remaining fish against *Y. ruckeri* (KC291153), the cause of red mouth disease in rainbow trout, was evaluated for 10 days. In summary, the fish from different treatments (*N* = 10) were transferred into 15 circular fiberglass tanks (150 L) located in the Research Station laboratory under the isolated and quarantined conditions. A dose of 1 × 10^7^ bacterial cells/ml [based on the median lethal dose (LD_50_) test] in PBS was prepared from *Y. ruckeri* using standard McFarland tubes, and the fish were injected intraperitoneally (0.1 ml per fish). The fish were fed with the experimental diets during the challenge text. The mortality and clinical signs of the infected fish were recorded daily. In addition, the standard bacteriological culture was performed on the kidney tissue samples of all dead fish to confirm the cause of death due to the pathogenic bacteria. The cumulative mortality was calculated on the basis of the following equation:

Cumulative mortality (%) = (total number of dead fish/ total number of stocked fish) × 100.

### Statistical Analysis

First, the homogeneity and normality of the data were investigated using the Levene's test and Kolmogorov–Smirnov test, respectively. Then, the data were analyzed by ANOVA using SPSS software (version 20). The mean comparison between different treatments was determined on the basis of the Tukey's multiple-range test at 5% probability level (*P* < 0.05).

## Results

### Total and Differential Leukocyte Counts

Cellular immune responses of rainbow trout at the end of the feeding trial are presented in Table 2. The WBC count in the groups fed with LE1 and LE2 diets was significantly higher than LE0.5 and the control groups (*P* < 0.05). In addition, Lym and Mono percentages in the fish fed with dietary LE2 were remarkably higher than those receiving the diet free from LE (control diet). Conversely, the lowest percentage of neutrophils belonged to the trout fed a diet containing LE2, which recorded a significant decrease compared with the control fish (*P* < 0.05). In the case of eosinophils, no significant differences were recorded between the different experimental groups (*P* > 0.05).

**Table 2:** Blood cell response of rainbow trout fed with diet containing different levels of licorice extract (LE) for 56 days^*^.

**Leukogram variables**	**Treatments (g kg** ^**−1**^ **)**				
	**Control**	**LE0.5**	**LE1**	**LE2**	**LE3**
WBC (×10^3^ μl^−1^)	21.55 ± 0.01^c^	25.61 ± 0.02^b^	25.80 ± 0.08^a^	25.83 ± 0.08^a^	25.69 ± 0.06^ab^
Neu (%)	17.00 ± 1.00^a^	16.33 ± 1.53^a^	16.33 ± 0.58^a^	12.00 ± 2.00^b^	15.33 ± 0.58^a^
Lym (%)	76.33 ± 1.53^b^	77 ± 1.00^b^	77.33 ± 1.53^b^	80.00 ± 1.00^a^	77.33 ± 1.53^b^
Mono (%)	5.33 ± 0.58^b^	6.33 ± 1.15^ab^	6.38 ± 0.58^ab^	7.67 ± 0.58^a^	7.00 ± 1.00^a^
Eos (%)	0.67 ± 0.58^a^	1.00 ± 0.66^a^	0.67 ± 0.58^a^	0.33 ± 0.58^a^	0.33 ± 0.85^a^

* *Different letters in each column show significant differences among the experimental groups (P < 0.05). Value are showed as mean ± SD (n = 3). WBC (g), white blood cells; Neu, neutrophils; Lym, lymphocytes; Mono, monocytes; Eos, eosinophils*.

### Serum Immune Parameters

**Figure 1:**
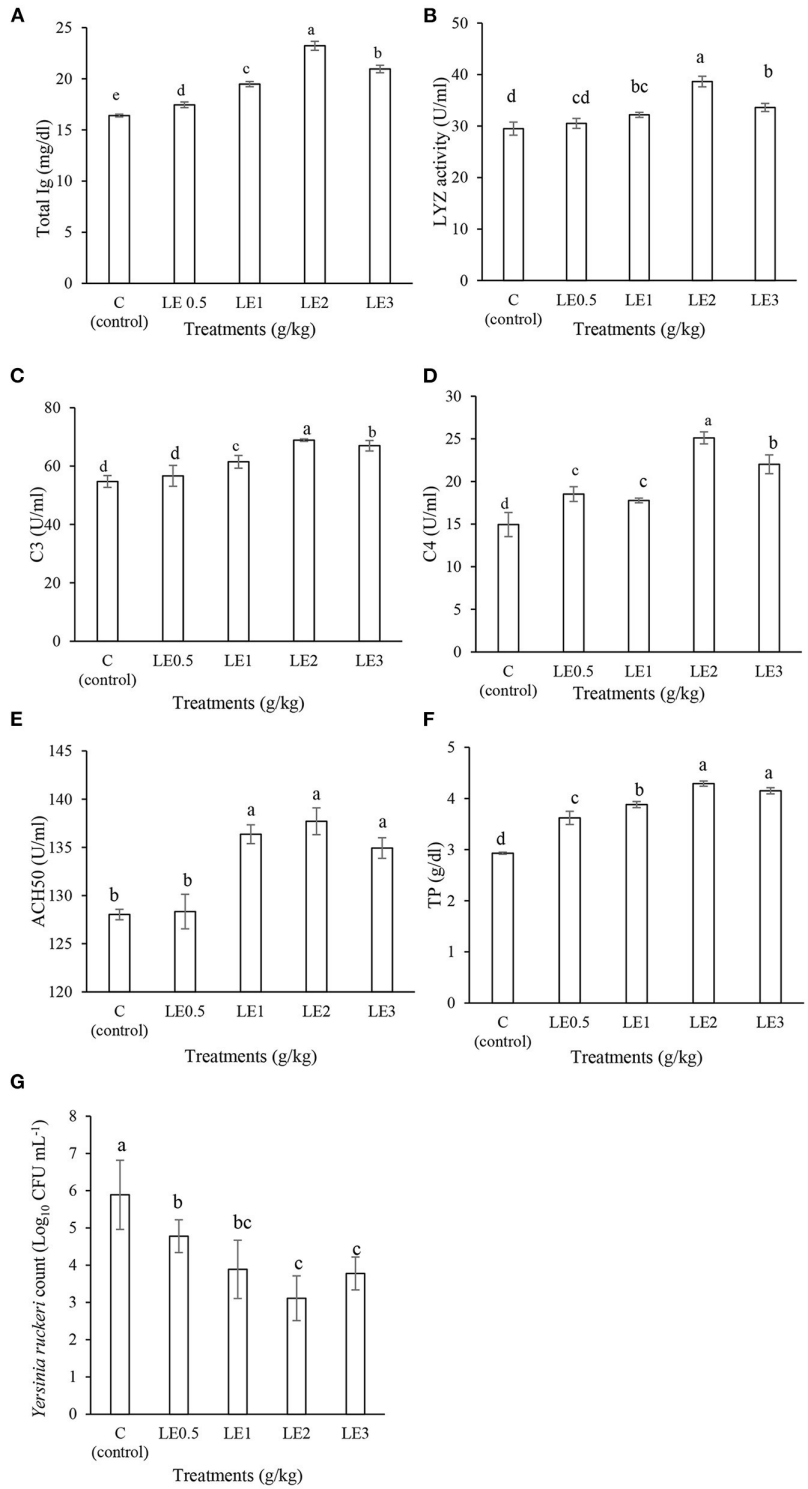
Evaluation of the serum immunological values of total immunoglobulin (Ig) **(A)**, lysozyme activity (LYZ) **(B)**, complement component 3 (C3) **(C)**, complement component 4 (C4) **(D)**, complement pathway hemolytic activity (ACH50) **(E)**, total protein (TP) **(F)**, and serum bacterial count **(G)** in rainbow trout fed with different levels of licorice extract (LE) supplemented diets. Bars assigned with the same superscripts are not significantly different (*P* > 0.05). Values are presented as the mean ± SD (*n* = 3).

As shown in Figure 1, the serum immune responses were affected by different levels of LE in rainbow trout. The fish fed with LE2 diet indicated significantly higher Ig, LYZ, C3, and C4 values than other treatments (*P* < 0.05). In addition, TP was remarkably higher in fish fed diets supplemented with LE2 and LE3 than other groups (*P* < 0.05). ACH50 activity was significantly enhanced in response to diets treated with LE1, LE2, and LE3, whereas no significant differences were observed between LE0.5 treatment and the control group (*P* > 0.05). Furthermore, the BA was significantly increased in the serum of the fish treated with dietary LE, and the strongest reduction of *Y. ruckeri* counts was recorded in the fish fed with LE2 and LE3 diets.

### Skin Mucus Immune Parameters

**Figure 2:**
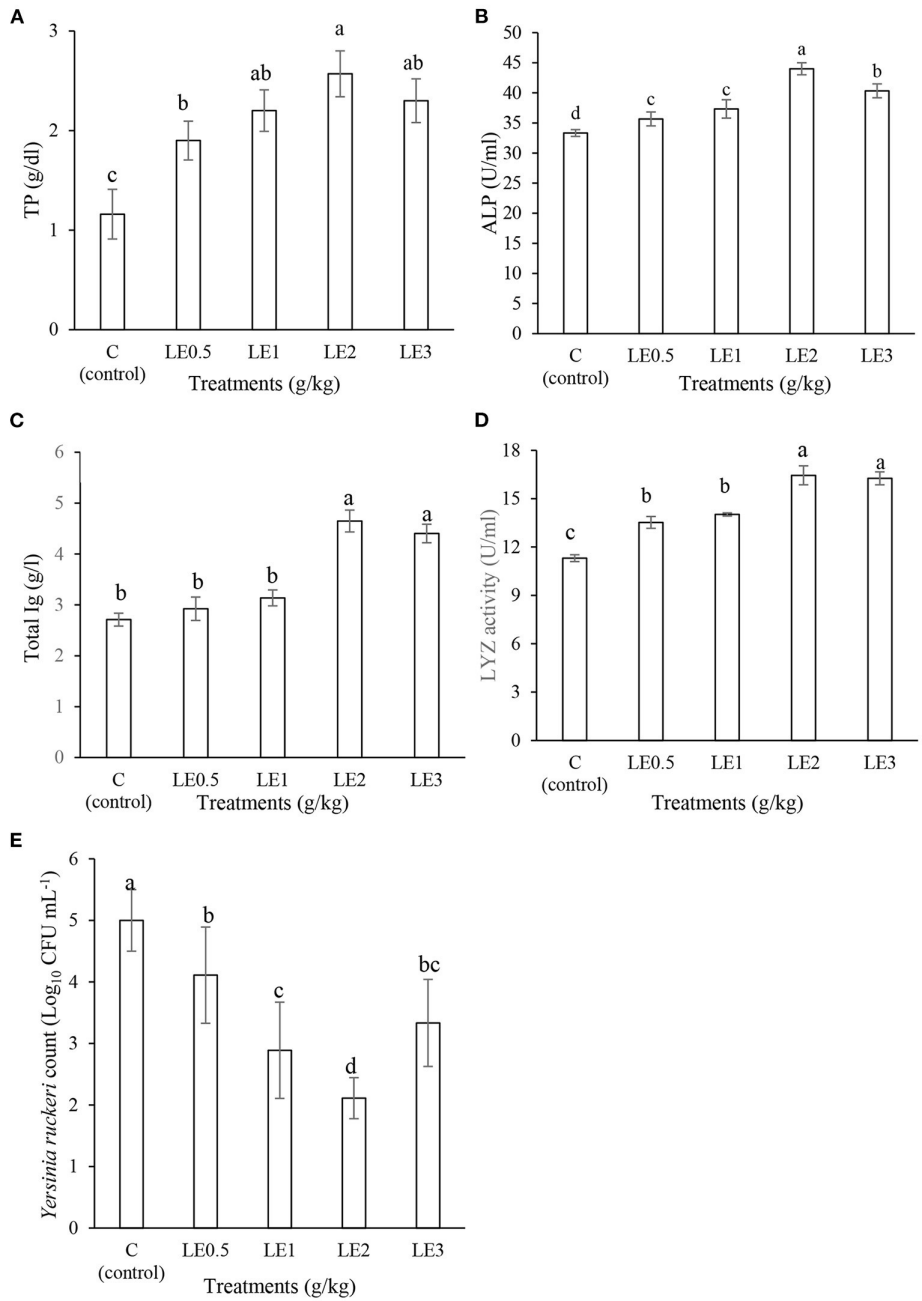
Evaluation of the skin mucosal immunological values of total protein (TP) **(A)**, alkaline phosphatase (ALP) **(B)**, total immunoglobulin (Ig) **(C)**, lysozyme activity (LYZ) **(D)**, and mucus bacterial count **(E)** in rainbow trout fed with different levels of licorice extract (LE) supplemented diets. Bars assigned with the same superscripts are not significantly different (*P* > 0.05). Values are presented as the mean ± SD (*n* = 3).

Figure 2 displays the effect of different levels of LE on the skin mucosal immunity of rainbow trout. The supplemented diets with LE2 and LE3 significantly enhanced the levels of LYZ activity, Ig, and TP values (*P* < 0.05). The highest level of ALP was determined in the fish treated with LE2 diet, and other treatments showed intermediate values and a significant difference compared with the control group (*P* < 0.05). The fish fed LE2 showed a higher value of mucus BA compared with other groups (*P* < 0.05). The control group, on the contrary, had the lowest mucus BA against *Y. ruckeri*.

### Immune-Related Gene Expression

**Figure 3:**
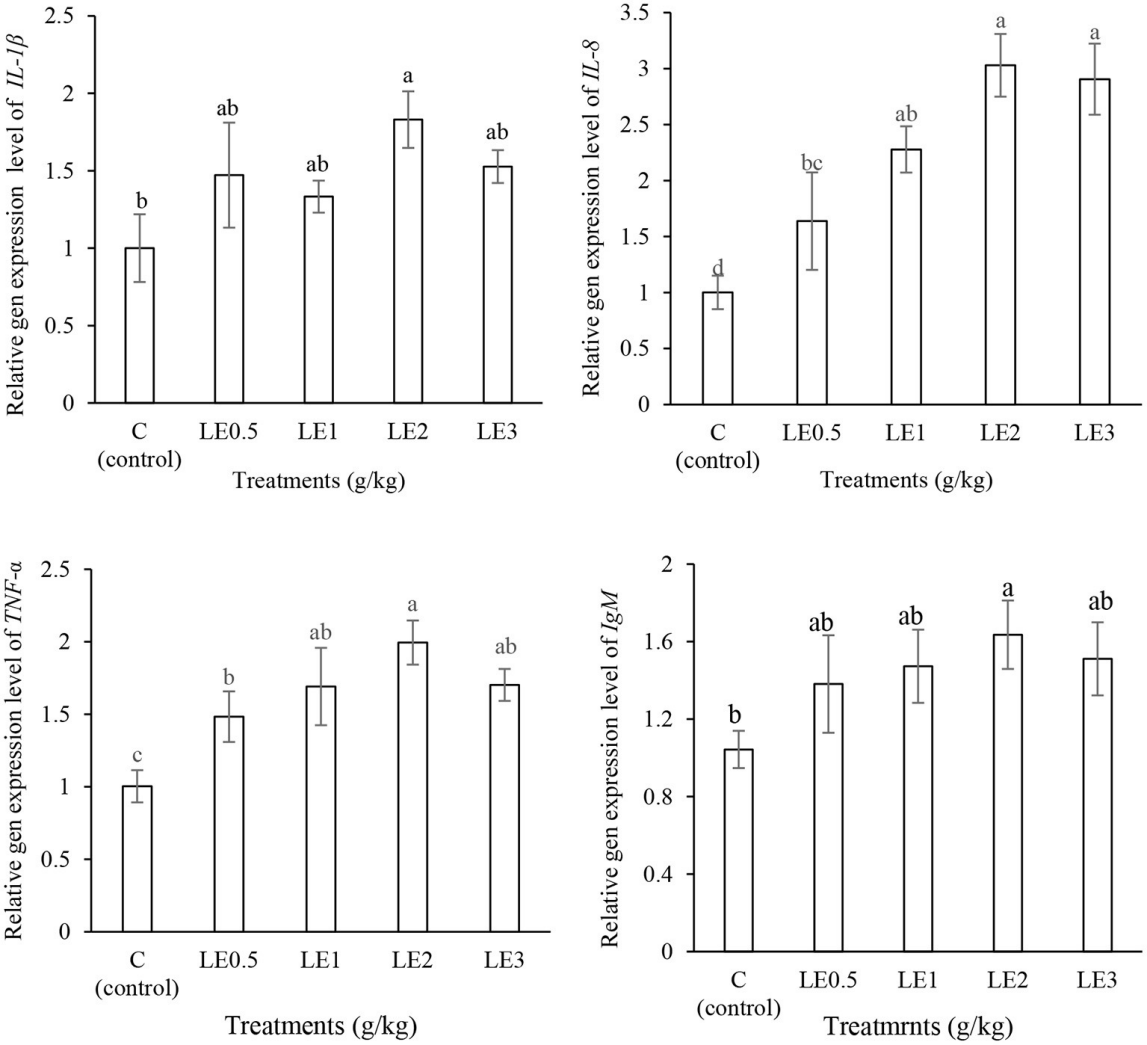
Measurement of the levels of interleukin-1β (*IL-1*β), interleukin-8 (*IL-8*), tumor necrosis factor α (*TNF-*α), and immunoglobulin M (*IgM*) gene expression in rainbow trout fed with different levels of licorice extract (LE) supplemented diets. Bars assigned with the same superscripts are not significantly different (*P* > 0.05). Values are presented as the mean ± SD (*n* = 3).

Expression of immune-related genes was significantly upregulated by LE dietary supplementation (Figure 3). The expression of *IL-1*β, *IgM*, and *TNF-*α genes was significantly increased in LE2 group (*P* < 0.05). In addition, the treated fish with LE2 and LE3 diets significantly indicated a higher level of *IL-8* gene expression, when compared with the control group and LE0.5 treatment (*P* < 0.05).

### Challenge Test

**Figure 4:**
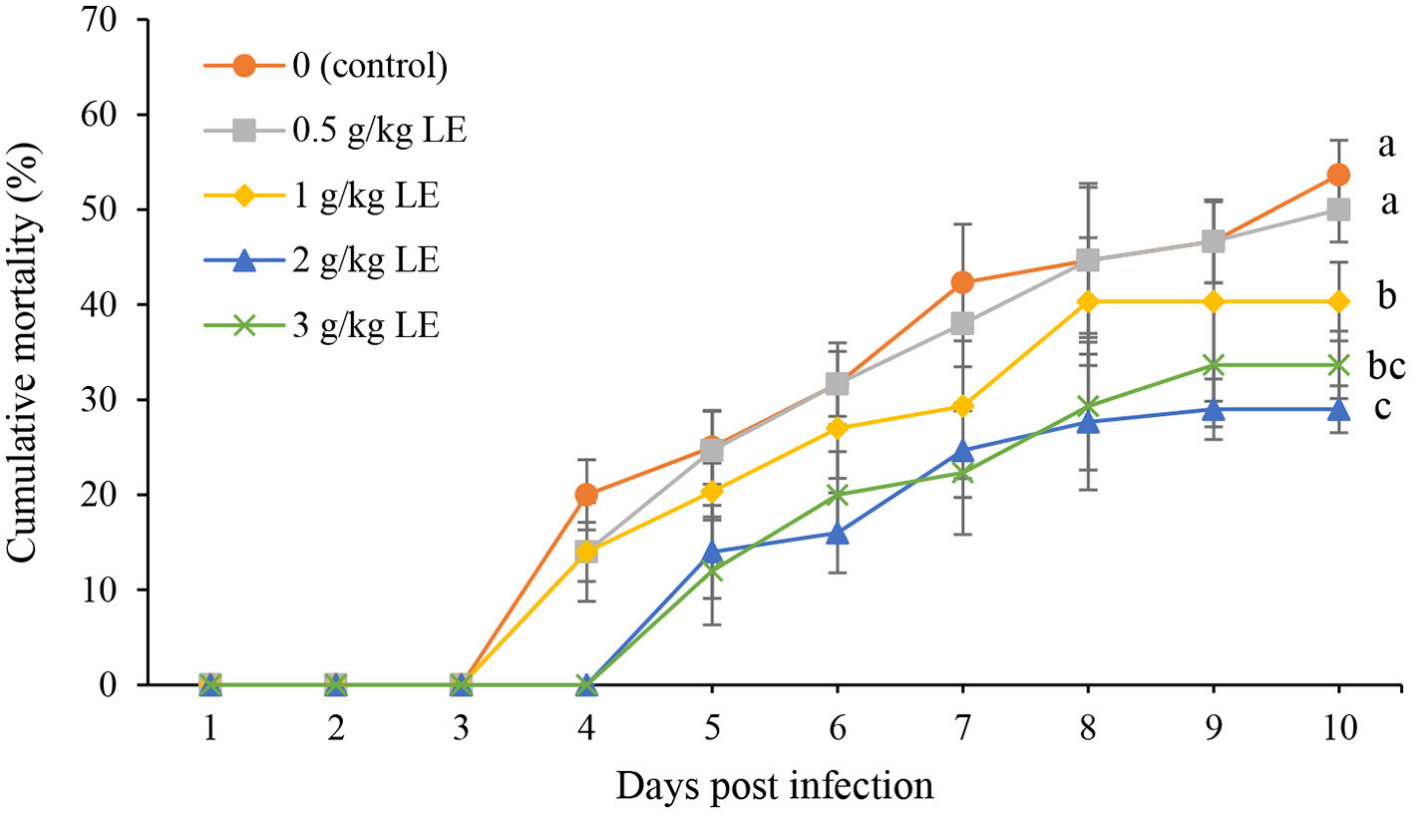
The cumulative mortality of rainbow trout fingerlings fed with different levels of licorice extract (LE) supplemented diets after experimentally challenged with *Yersinia ruckeri* for 10 days. Bars assigned with the same superscripts are not significantly different (*P* > 0.05). Values are presented as the mean ± SD (*n* = 3).

The trend of rainbow trout mortality during 10 days of experimentally challenged with *Y. ruckeri* infection is shown in Figure 4. A significant reduction (*P* < 0.05) in the mortality was observed in the fish fed with LE at higher concentrations than 0.5 g kg^−1^. In addition, the highest percentage of mortality was recorded in the control (53.66%) and 0.5 g kg^−1^ LE (50.00%) at the end of the bacterial challenge test. However, the mortality was started with a delay of 1 day (fourth day) in the groups of 2 and 3 g kg^−1^ LE. The lowest mortality was observed in the fish fed with LE2 diet (29%), which was not significantly different from LE3 treatment (33.66%; *P* > 0.05).

## Discussion

The application of feed supplements to improve the immune system of aquatic animals can reduce the economic losses due to infectious diseases outbreak in aquaculture sectors. In this regard, traditional plants are considered a common strategy to boost the immune system according to their remarkable antimicrobial, antioxidant, and therapeutic properties (3, 5, 7). Licorice is one of the most important medicinal plants and can be effective as a strong immunostimulant, which is also supported by our results on rainbow trout. However, the indicators of immunocompetence at the highest dietary LE level were slightly decreased compared with the fish belonging to LE2 treatment. This outcome may be associated with various factors such as the presence of anti-nutritional factors (saponin), allergic reactions, hyperglycemia, excessive excretion of potassium ions, and elevated plasma pH by consuming a high level of licorice (5, 20). However, finding the accurate mechanisms of the above topic in fish requires extensive studies in the future.

Improving leukocytes quantity and differential count is one of the vital parameters in the specific and non-specific immune system of fish that can be beneficially affected by adding herbal supplements in the diets (2, 4, 35, 36). On the basis of the results, the highest number of total leukocytes was observed in LE-added groups, which was significantly higher than the control group. The bioactive compounds in licorice (more than 20 triterpenoids and nearly 300 flavonoids), especially glycyrrhizin, 18β-glycyrrhetinic acid, licochalcone A and E, glabridin, and liquiritigenin, exert strong antimicrobial properties, which can modulate the immune system functions (37). Similar results were reported in great sturgeon, *Huso huso* (38) and striped catfish *Pangasianodon hypophthalmus* (35), when they were administrated orally with *Origanum vulgare* and *Phyllanthus amarus*, respectively. The main components of WBC engaged in the innate immune system are neutrophils, monocytes, macrophages, and eosinophils, which are potentially involved in bactericidal activities and phagocytosis (35). Unlike the innate mechanisms, B lymphocytes play a manifest role in specific immunity *via* producing antibodies against pathogens (36). The findings of the current study indicated that supplementation of rainbow trout with LE, especially at the level of 2 g kg^−1^, enhanced the lymphocyte and monocyte percentages, which can reveal the positive impacts of dietary LE in enhancing the defense system functions of rainbow trout. Moreover, our findings indicated that the neutrophils count has an inverse pattern with the lymphocytes. In agreement with our results, Rashmeei et al. (24) showed a decrease in the neutrophils count of goldfish (*Carassius auratus*) followed by an increase in dietary chasteberry (*Vitex agnus-castus*) extract concentration. Neutrophils and eosinophils are motile cells that accumulate at infectious sites and kill pathogens by producing O^2−^ and OH^−^ ions through the process of respiratory burst (39). However, we could not find a strong explanation for the role of LE on the neutrophils. Therefore, further studies are needed to elucidate the dietary effect of LE on fish neutrophils.

The humoral immune system has many molecules with diverse activities. For instance, the complement system contains 35 types of soluble proteins that play a vital role in chemotaxis, inflammatory, and phagocytic responses (40). Besides, LYZ and Ig are two other known components of humoral immunity that are involved in bacteria wall lysis and virus neutralization, respectively (36). In our study, rainbow trout fed with LE2 diet indicated higher values of serum LYZ, C3, C4, ACH50, and Ig than the control fish. Similarly, Abdel-Tawwab and El-Araby (19) reported that Nile tilapia fed the diet treated with 20 g kg^−1^ licorice powder remarkably increased serum LYZ activity. In addition, Adineh et al. (18) elucidated higher serum LYZ and ACH50 activities as well as Ig value in common carp fed diets enriched with different levels of licorice powder. Improving liver function by licorice root is one of the main reasons for the increased serum TP, C3, C4, and ACH50 levels that were previously suggested by Elabd et al. (20) and Yin et al. (21). These findings may be related to the role of LE to improve liver and other organs functions that produced serum proteins (18). Earlier reports indicated that dietary administration of licorice (main active compound: glycyrrhizin) can decrease liver damages due to the hepatoprotective and antioxidant effects of *G. glabra* in fish (19, 20). In fact, the protective effect of licorice against liver fibrosis and cirrhosis may be related to its anti-inflammatory activity and enhancement of antioxidant defense in the host (41). In addition, an increase in the serum TP content can be accompanied by increasing in the Ig and total globulin levels (17). Moreover, it seems that increasing the number of lymphocytes could beneficially affect the level of LYZ activity and Ig value of rainbow trout (42, 43). In this study, the highest level of serum BA activity was recorded in the fish fed LE2 diet by observing the lowest *Y. ruckeri* count. This can be interpreted by improving the integrity of the mucosal tissues due to the phenolic compounds (mainly glycosides of liquiritigenin and isoliquiritigenin) found in LE (44). Similarly, previous studies have also confirmed that enhancing serum immune responses by diets containing plant extracts such as *Taraxacum officinale* (45), *Viscum album* (46), *Mentha piperita* (47), and *Ziziphora clinopodioides* (48) led to increase serum BA in rainbow trout.

The skin and gills of fish are constantly exposed to destructive agents such as pathogens, toxins, and stressors (49, 50). Therefore, improving their protective layer, such as epidermal mucus, is crucial in the first line of defense against invading pathogens. Skin mucosal barrier in the fish defense contains a variety of biomolecules such as lectins, peptides, LYZ, immunoglobulin, and proteolytic enzymes (44, 45). In recent years, several nutritional studies have been performed to improve mucosal immunity (51–53). The possible mechanism of immunostimulants in the regulation of mucosal immune parameters is through stimulation or contact with lymphatic tissue related to the skin or gills (51). Several studies have shown that the oral administration of medicinal plants and their derivatives stimulate lymph tissues to secrete more epidermal mucus defense elements (53–55). Our findings showed that dietary supplementation with different levels of LE, especially at 2 and 3 g kg^−1^, resulted in a significant improvement in LYZ and ALP activities as well as Ig value compared with the control group. These findings were supported by previous reports in rainbow trout fed with other medicinal plants. For instance, Gholamhosseini et al. (56) showed that dietary Tarragon (*Artemisia dracunculus*) improved LYZ and ALP activity and TP value in rainbow trout. In another study, Ghafarifarsani et al. (57) indicated that rainbow trout fed diet enriched with oak acorn (*Quercus liaotungensis*) extract had remarkably higher skin mucus LYZ and ALP activities compared with the control group. The beneficial effects of licorice on the immune responses may be due to immunomodulatory compounds such as glycyrrhizin, which was previously reported (18–20). The present findings on the mucus BA confirmed that LE can improve skin mucus immune parameters in rainbow trout as a potential source of antibacterial substances. Similarly, Oroji et al. (48) reported that oral administration of *Ziziphora clinopodioides* extract significantly increased the BA of skin mucus in rainbow trout. In addition, Ghafarifarsani et al. (57) reported the strong antibacterial effect of skin mucus extract against *Y. ruckeri* in rainbow trout treated by dietary oak acorn extract.

Fish immune systems are complex and significantly influenced by nutritional status (10, 58). In the context of immune and inflammatory responses, cytokines are soluble glycoproteins that play a major role in regulating immune responses and can be divided into various groups such as interferons, interleukins, tumor necrosis factor, and chemokines (59, 60). Several studies have suggested that the expression of pro-inflammatory cytokines genes (*IL-1*β, *IL-6, IL-8*, and *TNF-*α) was upregulated by plant-fortified diets such as *Ginkgo biloba* (61)*, Phoenix dactylifera* (62), *Origanum vulgare* (4), and *Taraxacum officinale* (45). In this study, the expression of *TNF-*α and *IL-8* genes significantly increased in response to different levels of LE, especially at 2 g kg^−1^. In fact, *TNF-*α is involved in initiating and enhancing inflammatory processes against gram-negative bacteria and other pathogens (63). In addition, pro-inflammatory cytokines such as *IL-8* play a fundamental role in the acute inflammatory process, neutrophil oxidative burst, and wound healing (64). In the present study, the expression of *IL-1*β and *IgM* genes significantly increased with the increase of dietary LE and peaked at LE2; however, thereafter, they were slightly decreased. *IL-1*β plays a key role in systemic responses to infections and tissue injuries, and it has a similar function with *TNF-*α and enhances the activity of LYZ and cytokines with antibacterial properties such as *IL-17* (65). To our knowledge, no data are available on the effect of licorice on the expression of immune-related genes of rainbow trout or other species. However, a pair of studies showed that the supplementation of rainbow trout diet with *Polygonum minus* (66) and *Aloysia citrodora* (67) could upregulate the expression of *TNF-*α, *IL-1*β, and *IL8* genes. In this study, the expression of pro-inflammatory cytokine genes was elevated in LE-added groups, especially at 2 g kg^−1^, which may indicate the importance of phytogenic bioactive compounds in LE to modulate immune responses in rainbow trout.

The efficacy of dietary supplements can be evaluated in aquaculture by exposing the fish to pathogens. *Y. ruckeri* is the cause of enteric red mouth disease or yersiniosis, which is a septicemic infection that affects salmonids, and rainbow trout is more susceptible to this infection at early life stages (68). This infectious disease is widespread and has caused significant economic losses in the trout aquaculture industry (69). In the present study, the infected rainbow trout with *Y. ruckeri* indicated a significantly lower mortality rate in the LE-added groups, especially in LE2 treatment, compared with the control group. The positive effect of various medicinal plants in increasing the survival rate of rainbow trout against yersiniosis has been previously reported (23, 57, 66, 70, 71). The increase in the survival and resistance of rainbow trout against *Y. ruckeri* infection in LE 2 and LE3 treatments can confirm the enhancement of the fish innate immunity and health, which are in line with the improvements in the measured immune parameters of serum and skin mucus.

Our findings revealed that using LE as a feed additive can enhance innate immune responses in rainbow trout. In this study, a diet containing LE at 2 g kg^−1^ effectively improved the leukocyte count, serum, and mucus immune responses as well as some immune-mediated genes in the head kidney. The oral administration of LE, especially at 2 g kg^−1^ increased the resistance of fish to *Y. ruckeri* infection. Therefore, it seems that the use of LE in rainbow trout diet as an immunomodulatory agent can be effective to boost the immune system.

## Data Availability Statement

The raw data supporting the conclusions of this article will be made available by the authors, without undue reservation.

## Ethics Statement

All the protocols were approved by the Science and Research Branch University, Committee of Faculty of Natural Resources and Environment, Tehran, Iran (Local Approval No. 162409789, 03/15/2020).

## Author Contributions

MD carried out fish maintenance and sample collection. MS contributed to conception and design of the study. AK organized the database and performed the statistical analysis. All authors read, wrote, and approved the submitted manuscript version.

## Funding

This study is a part of a Ph.D. thesis in aquaculture and supported by the University of Science and Research Branch (IAU, Tehran, Iran).

## Conflict of Interest

The authors declare that the research was conducted in the absence of any commercial or financial relationships that could be construed as a potential conflict of interest.

## Publisher's Note

All claims expressed in this article are solely those of the authors and do not necessarily represent those of their affiliated organizations, or those of the publisher, the editors and the reviewers. Any product that may be evaluated in this article, or claim that may be made by its manufacturer, is not guaranteed or endorsed by the publisher.

## References

[1] Zhu F. A review on the application of herbal medicines in the disease control of aquatic animals. Aquaculture. (2020) 526:735422. 10.1016/j.aquaculture.2020.735422

[2] Awad E Awaad A. Role of medicinal plants on growth performance and immune status in fish. Fish Shellfish Immunol. (2017) 67:40–54. 10.1016/j.fsi.2017.05.034 28526570

[3] Reverter M Bontemps N Lecchini D Banaigs B Sasal P. Use of plant extracts in fish aquaculture as an alternative to chemotherapy: current status and future perspectives. Aquaculture. (2014) 433:50–61. 10.1016/j.aquaculture.2014.05.048

[4] Abdel-Latif HM Abdel-Tawwab M Khafaga AF. Dawood MA. Dietary origanum essential oil improved antioxidative status, immune-related genes, and resistance of common carp (*Cyprinus carpio* L.) to Aeromonas hydrophila infection Fish. Shellfish Immunol. (2020) 104:1–7. 10.1016/j.fsi.2020.05.056 32474085

[5] Gabriel NN. Review on the progress in the role of herbal extracts in tilapia culture. Cogent Food Agri. (2019) 5:1619651. 10.1080/23311932.2019.1619651

[6] Wang W Sun J Liu C Xue Z. Application of immunostimulants in aquaculture: current knowledge and future perspectives. Aquac Res. (2017) 48:1–23. 10.1111/are.13161

[7] Shekarabi SPH Javarsiani L Mehrgan MS Dawood MA Adel M. Growth performance, blood biochemistry profile, and immune response of rainbow trout (*Oncorhynchus mykiss*) fed dietary Persian shallot (*Allium stipitatum*) powder. Aquaculture. (2021) 548:737627. 10.1016/j.aquaculture.2021.737627

[8] Dawood MA Koshio S Esteban MÁ. Beneficial roles of feed additives as immunostimulants in aquaculture: a review. Rev Aquacult. (2018) 10:950–74. 10.1111/raq.12209

[9] Van Hai N. The use of medicinal plants as immunostimulants in aquaculture: a review. Aquaculture. (2015) 446:88–96. 10.1016/j.aquaculture.2015.03.014

[10] Galina J Yin G Ardo L Jeney Z. The use of immunostimulating herbs in fish. An overview of research Fish. Physiol Biochem. (2009) 35:669–76. 10.1007/s10695-009-9304-z 19277888

[11] Shankar K Kiran B. Review on usage of medicinal plants in fish diseases. Int J Pharma Bio Sci. (2013) 4:975–86.34164770

[12] Stratev D Zhelyazkov G Noundou XS Krause RW. Beneficial effects of medicinal plants in fish diseases. Aquacult Int. (2018) 26:289–308. 10.1007/s10499-017-0219-x

[13] Pastorino G Cornara L Soares S Rodrigues F Oliveira MBP. Liquorice (*Glycyrrhiza glabra*): a phytochemical and pharmacological review. Phytother Res. (2018) 32:2323–39. 10.1002/ptr.6178 30117204 PMC7167772

[14] Alagawany M Elnesr SS Farag MR El-Hack A Mohamed E Khafaga AF. Use of licorice (*Glycyrrhiza glabra*) herb as a feed additive in poultry: current knowledge and prospects. Animals. (2019) 9:536. 10.3390/ani9080536 31394812 PMC6720273

[15] Karkanis A Martins N Petropoulos S. Ferreira IC. Phytochemical composition, health effects, and crop management of liquorice (*Glycyrrhiza glabra* L.): A medicinal plant. Food Rev Int. (2018) 34:182–203. 10.1080/87559129.2016.1261300

[16] Mamedov NA Egamberdieva D. Phytochemical constituents and pharmacological effects of licorice: a review. Plant Human Health. (2019) 3:1–21. 10.1007/978-3-030-04408-4_131811935

[17] Yang R Yuan B-C Ma Y-S Zhou S Liu Y. The anti-inflammatory activity of licorice, a widely used Chinese herb. Pharm Biol. (2017) 55:5–18. 10.1080/13880209.2016.1225775 27650551 PMC7012004

[18] Adineh H Naderi M Yousefi M Khademi Hamidi M Ahmadifar E Hoseini SM. Dietary licorice (*Glycyrrhiza glabra*) improves growth, lipid metabolism, antioxidant and immune responses, and resistance to crowding stress in common carp, *Cyprinus carpio*. Aquaculture Nutr. (2020) 27:417–26. 10.1111/anu.13194

[19] Abdel-Tawwab M El-Araby DA. Immune and antioxidative effects of dietary licorice (Glycyrrhiza glabra L) on performance of Nile tilapia, Oreochromis niloticus (L) and its susceptibility to Aeromonas hydrophila infection. Aquaculture. (2021) 530:735828. 10.1016/j.aquaculture.2020.735828

[20] Elabd H Wang H-P Shaheen A Yao H Abbass A. Feeding *Glycyrrhiza glabra* (liquorice) and Astragalus membranaceus (AM) alters innate immune and physiological responses in yellow perch (*Perca flavescens*). Fish Shellfish Immunol. (2016) 54:374–84. 10.1016/j.fsi.2016.04.024 27129627

[21] Yin G Cao L Xu P Jeney G Nakao M Lu C. Hepatoprotective and antioxidant effects of *Glycyrrhiza glabra* extract against carbon tetrachloride (CCl 4)-induced hepatocyte damage in common carp (*Cyprinus carpio*). Fish Physiol Biochem. (2011) 37:209–16. 10.1007/s10695-010-9436-1 20865324

[22] Isbrucker R Burdock G. Risk and safety assessment on the consumption of Licorice root (Glycyrrhiza sp), its extract and powder as a food ingredient, with emphasis on the pharmacology and toxicology of glycyrrhizin. Regul Toxicol Pharmacol. (2006) 46:167–92. 10.1016/j.yrtph.2006.06.002 16884839

[23] Farsani MN Hoseinifar SH Rashidian G Farsani HG Ashouri G Van Doan H. Dietary effects of Coriandrum sativum extract on growth performance, physiological and innate immune responses and resistance of rainbow trout (*Oncorhynchus mykiss*) against *Yersinia ruckeri*. Fish Shellfish Immunol. (2019) 91:233–40. 10.1016/j.fsi.2019.05.031 31102711

[24] Rashmeei M Shekarabi SPH Mehrgan MS Paknejad H. Stimulatory effect of dietary chasteberry (*Vitex agnus-castus*) extract on immunity, some immune-related gene expression, and resistance against Aeromonas hydrophila infection in goldfish (*Carassius auratus*). Fish Shellfish Immunol. (2020) 107:129–36. 10.1016/j.fsi.2020.09.037 33002603

[25] Martins ML Nomura DT Myiazaki DMY Pilarsky F Ribeiro K de Castro MP. Physiological and haematological response of *Oreochromis niloticus* (Osteichthyes: Cichlidae) exposed to single and consecutive stress of capture. Acta Scientiarum Animal Sci. (2004) 26:449–56. 10.4025/actascianimsci.v26i4.1719

[26] Borges A Scotti LV Siqueira DR Jurinitz DF Wassermann GF. Hematologic and serum biochemical values for jundiá (*Rhamdia quelen*). Fish Physiol Biochem. (2004) 30:21–5. 10.1007/s10695-004-5000-1

[27] Lowry OH Rosebrough NJ Farr AL Randall RJ. Protein measurement with the Folin phenol reagent. J Biol Chem. (1951) 193:265–75. 10.1016/S0021-9258(19)52451-6 14907713

[28] Siwicki A Anderson D. Immunoglobulin Levels in Fish Sera Measured by Polyethylene Glycol and Spectrophotometric Methods in Microtiter Plates. Fair Haven: Techniques in fish immunology III SOS Publications (1994).

[29] Ellis AE. Serum antiproteases in fish. In: Stolen JS Fletcher TC Anderson DP Hattari SC Rowley AF editors. Techniques in Fish Immunology. New Jersey, NJ: SOS Publications (1990). pp. 95–99.

[30] Yano T. Assay of hemolytic complement activity. In: Stolen JS Fletcher TC Anderson DP Hattari SC Rowley AF editors. Techniques in Fish Immunology. New Jersey, NJ: SOS Publications (1990). pp. 131–141.

[31] Gheytasi A Shekarabi SPH Islami HR Mehrgan MS. Feeding rainbow trout, *Oncorhynchus mykiss*, with lemon essential oil loaded in chitosan nanoparticles: effect on growth performance, serum hemato-immunological parameters, and body composition. Aquaculture Int. (2021) 29:2207–21. 10.1007/s10499-021-00741-2

[32] Fazelan Z Vatnikov YA Kulikov EV Plushikov VG Yousefi M. Effects of dietary ginger (*Zingiber officinale*) administration on growth performance and stress, immunological, and antioxidant responses of common carp (*Cyprinus carpio*) reared under high stocking density. Aquaculture. (2020) 518:734833. 10.1016/j.aquaculture.2019.734833

[33] García-Torrico AI Guijarro JA Cascales D Méndez J. Changes in physiology and virulence during the selection of resistant *Yersinia ruckeri* mutants under subinhibitory cefotaxime concentrations. J Fish Dis. (2019) 42:1687–96. 10.1111/jfd.13086 31617230

[34] Wiame I Remy S Swennen R Sági L. Irreversible heat inactivation of DNase I without RNA degradation. Biotechniques. (2000) 29:252–6. 10.2144/00292bm11 10948426

[35] Nhu TQ Hang BTB Vinikas A Hue BTB Quetin-Leclercq J Scippo M-L. Screening of immuno-modulatory potential of different herbal plant extracts using striped catfish (*Pangasianodon hypophthalmus*) leukocyte-based in vitro tests. Fish Shellfish Immunol. (2019) 93:296–307. 10.1016/j.fsi.2019.07.064 31352112

[36] Magnadóttir B. Innate immunity of fish (overview). Fish Shellfish Immunol. (2006) 20:137–51. 10.1016/j.fsi.2004.09.006 15950491

[37] Wang L Yang R Yuan B Liu Y Liu C. The antiviral and antimicrobial activities of licorice, a widely-used Chinese herb. Acta Pharmaceutica Sinica B. (2015) 5:310–5. 10.1016/j.apsb.2015.05.005 26579460 PMC4629407

[38] Ahmadifar E Mansour MR Amirkolaie AK Rayeni MF. Growth efficiency, survival and haematological changes in great sturgeon (*Huso huso* Linnaeus, 1758) juveniles fed diets supplemented with different levels of thymol–carvacrol. Anim Feed Sci Technol. (2014) 198:304–8. 10.1016/j.anifeedsci.2014.08.012

[39] Dorucu M Ispir U Colak S Altinterim B Celayir Y. The effect of black cumin seeds, Nigella sativa, on the immune response of rainbow trout, *Oncorhynchus mykiss*. Mediterr Aquaculture J. (2009) 2:27–33. 10.21608/maj.2009.2667

[40] Sunyer J Boshra H Li J. Evolution of anaphylatoxins, their diversity and novel roles in innate immunity: insights from the study of fish complement. Vet Immunol Immunopathol. (2005) 108:77–89. 10.1016/j.vetimm.2005.07.009 16112742

[41] Jung J-C Lee Y-H Kim SH Kim K-J Kim K-M Oh S. Hepatoprotective effect of licorice, the root of *Glycyrrhiza uralensis* Fischer, in alcohol-induced fatty liver disease. BMC Complement Altern Med. (2015) 16:1–10. 10.1186/s12906-016-0997-0 26801973 PMC4722619

[42] Ebrahimi E Haghjou M Nematollahi A Goudarzian F. Effects of rosemary essential oil on growth performance and hematological parameters of young great sturgeon (*Huso huso*). Aquaculture. (2020) 521:734909. 10.1016/j.aquaculture.2019.734909

[43] Ghodrati M Islami HR Shekarabi SPH Masouleh AS Mehrgan MS. Combined effects of enzymes and probiotics on hemato-biochemical parameters and immunological responses of juvenile Siberian sturgeon (*Acipenser baerii*). Fish Shellfish Immunol. (2021) 112:116–24. 10.1016/j.fsi.2021.03.003 33713825

[44] Firmino JP Galindo-Villegas J Reyes-López FE Gisbert E. Phytogenic bioactive compounds shape fish mucosal immunity. Front Immunol. (2021) 12:695973. 10.3389/fimmu.2021.695973 34220858 PMC8252966

[45] Shekarabi SPH Mostafavi ZS Mehrgan MS Islami HR. Dietary supplementation with dandelion (*Taraxacum officinale*) flower extract provides immunostimulation and resistance against Streptococcus iniae infection in rainbow trout (*Oncorhynchus mykiss*). Fish Shellfish Immunol. (2021) 118:180–7. 10.1016/j.fsi.2021.09.004 34506883

[46] Yousefi M Farsani MN Ghafarifarsani H Hoseinifar SH Van Doan H. The effects of dietary supplementation of mistletoe (*Viscum album*) extract on the growth performance, antioxidant, and innate, immune responses of rainbow trout (*Oncorhynchus mykiss*). Aquaculture. (2021) 536:736385. 10.1016/j.aquaculture.2021.736385

[47] Abdel-Tawwab M Abbass FE. Turmeric powder, Curcuma longa L, in common carp, Cyprinus carpio L, diets: growth performance, innate immunity, and challenge against pathogenic Aeromonas hydrophila infection. J World Aquaculture Soc. (2017) 48:303–12. 10.1111/jwas.12349

[48] Oroji E Mehrgan MS Islami HR Sharifpour I. Dietary effect of *Ziziphora clinopodioides* extract on zootechnical performance, immune response, and disease resistance against *Yersinia ruckeri* in *Oncorhynchus mykiss*. Aquaculture Rep. (2021) 21:100827. 10.1016/j.aqrep.2021.100827

[49] Guardiola FA Cuesta A Abellán E Meseguer J Esteban MA. Comparative analysis of the humoral immunity of skin mucus from several marine teleost fish. Fish Shellfish Immunol. (2014) 40:24–31. 10.1016/j.fsi.2014.06.018 24972341

[50] Reverter M Tapissier-Bontemps N Lecchini D Banaigs B Sasal P. Biological and ecological roles of external fish mucus: a review. Fishes. (2018) 3:41. 10.3390/fishes3040041

[51] Salinas I. The mucosal immune system of teleost fish. Biology. (2015) 4:525–39. 10.3390/biology4030525 26274978 PMC4588148

[52] Paknejad H Shekarabi SPH Mehrgan MS Hajimoradloo A Khorshidi Z Rastegari S. Dietary peppermint (*Mentha piperita*) powder affects growth performance, hematological indices, skin mucosal immune parameters, and expression of growth and stress-related genes in Caspian roach (*Rutilus caspicus*). Fish Physiol Biochem. (2020) 46:1883–95. 10.1007/s10695-020-00839-z 32592128

[53] Hoseinifar SH Khalili M Rufchaei R Raeisi M Attar M Cordero H. Effects of date palm fruit extracts on skin mucosal immunity, immune related genes expression and growth performance of common carp (*Cyprinus carpio*) fry. Fish Shellfish Immunol. (2015) 47:706–11. 10.1016/j.fsi.2015.09.046 26439417

[54] Harikrishnan R Balasundaram C Heo M-S. Diet enriched with mushroom *Phellinus linteus* extract enhances the growth, innate immune response, and disease resistance of kelp grouper, *Epinephelus bruneus* against vibriosis. Fish Shellfish Immunol. (2011) 30:128–34. 10.1016/j.fsi.2010.09.013 20883799

[55] Taee HM Hajimoradloo A Hoseinifar SH. Ahmadvand H. Dietary Myrtle (*Myrtus communis* L.) improved non-specific immune parameters and bactericidal activity of skin mucus in rainbow trout (Oncorhynchus mykiss) fingerlings. Fish Shellfish Immunol. (2017) 64:320–4. 10.1016/j.fsi.2017.03.034 28330807

[56] Gholamhosseini A Hosseinzadeh S Soltanian S Banaee M Sureda A Rakhshaninejad M. Effect of dietary supplements of *Artemisia dracunculus* extract on the haemato-immunological and biochemical response, and growth performance of the rainbow trout (*Oncorhynchus mykiss*). Aquac Res. (2020) 52:2097–2109. 10.1111/are.15062

[57] Ghafarifarsani H Rashidian G Sheikhlar A Naderi Farsani M Hoseinifar SH Van Doan H. The use of dietary oak acorn extract to improve haematological parameters, mucosal and serum immunity, skin mucus bactericidal activity, and disease resistance in rainbow trout (*Oncorhynchus mykiss*). Aquac Res. (2021) 52:2518–27. 10.1111/are.15101

[58] Harikrishnan R Balasundaram C Heo M-S. Impact of plant products on innate and adaptive immune system of cultured finfish and shellfish. Aquaculture. (2011) 317:1–15. 10.1016/j.aquaculture.2011.03.039

[59] Wang T Secombes CJ. The cytokine networks of adaptive immunity in fish. Fish Shellfish Immunol. (2013) 35:1703–18. 10.1016/j.fsi.2013.08.030 24036335

[60] Rimoldi S Finzi G Ceccotti C Girardello R Grimaldi A Ascione C. Butyrate and taurine exert a mitigating effect on the inflamed distal intestine of European sea bass fed with a high percentage of soybean meal. Fish Aquat Sci. (2016) 19:1–14. 10.1186/s41240-016-0041-9

[61] Bao L Chen Y Li H Zhang J Wu P Ye K. Dietary *Ginkgo biloba* leaf extract alters immune-related gene expression and disease resistance to Aeromonas hydrophila in common carp *Cyprinus carpio*. Fish Shellfish Immunol. (2019) 94:810–8. 10.1016/j.fsi.2019.09.056 31546037

[62] Guardiola F Porcino C Cerezuela R Cuesta A Faggio C Esteban M. Impact of date palm fruits extracts and probiotic enriched diet on antioxidant status, innate immune response and immune-related gene expression of European seabass (*Dicentrarchus labrax*). Fish Shellfish Immunol. (2016) 52:298–308. 10.1016/j.fsi.2016.03.152 27033470

[63] Feng L Li W Liu Y Jiang W-D Kuang S-Y Jiang J. Dietary phenylalanine-improved intestinal barrier health in young grass carp (*Ctenopharyngodon idella*) is associated with increased immune status and regulated gene expression of cytokines, tight junction proteins, antioxidant enzymes and related signalling molecules. Fish Shellfish Immunol. (2015) 45:495–509. 10.1016/j.fsi.2015.05.001 25979603

[64] Li C Yao C-L. Molecular and expression characterizations of interleukin-8 gene in large yellow croaker (*Larimichthys crocea*). Fish Shellfish Immunol. (2013) 34:799–809. 10.1016/j.fsi.2012.12.019 23333360

[65] Hong S Peddie S Campos-Pérez JJ Zou J Secombes CJ. The effect of intraperitoneally administered recombinant IL-1β on immune parameters and resistance to *Aeromonas salmonicida* in the rainbow trout (*Oncorhynchus mykiss*). Develop Compar Immunol. (2003) 27:801–12. 10.1016/S0145-305X(03)00056-9 12818637

[66] Adel M Dawood MA Shafiei S Sakhaie F Shekarabi SPH. Dietary Polygonum minus extract ameliorated the growth performance, humoral immune parameters, immune-related gene expression and resistance against Yersinia ruckeri in rainbow trout (*Oncorhynchus mykiss*). Aquaculture. (2020) 519:734738. 10.1016/j.aquaculture.2019.734738

[67] Hoseinifar SH Shakouri M Van Doan H Shafiei S Yousefi M Raeisi M. Dietary supplementation of lemon verbena (*Aloysia citrodora*) improved immunity, immune-related genes expression and antioxidant enzymes in rainbow trout (*Oncorrhyncus mykiss*). Fish Shellfish Immunol. (2020) 99:379–85. 10.1016/j.fsi.2020.02.006 32032763

[68] Fernández L Méndez J Guijarro JA. Molecular virulence mechanisms of the fish pathogen Yersinia ruckeri. Vet Microbiol. (2007) 125:1–10. 10.1016/j.vetmic.2007.06.013 17651924

[69] Wrobel A Leo JC Linke D. Overcoming fish defences: the virulence factors of *Yersinia ruckeri*. Genes. (2019) 10:700. 10.3390/genes10090700 31514317 PMC6770984

[70] Adel M Pourgholam R Zorriehzahra J Ghiasi M. Hemato–Immunological and biochemical parameters, skin antibacterial activity, and survival in rainbow trout (*Oncorhynchus mykiss*) following the diet supplemented with *Mentha piperita* against *Yersinia ruckeri*. Fish Shellfish Immunol. (2016) 55:267–73. 10.1016/j.fsi.2016.05.040 27245867

[71] Soltanian M Langrodi HF Nejad MM. The use of *Zingiber officinale* extract against *Yersinia ruckeri* and its effects on the antioxidant status and immune response in *Oncorhynchus mykiss*. Int J Aquat Biol. (2019)7:301–14. 10.22034/ijab.v7i5.630

